# Dental cleaning behavior and related factors among hemodialysis patients in the West of Iran: A cross‐sectional study

**DOI:** 10.1002/cre2.745

**Published:** 2023-05-03

**Authors:** Arezoo Fallahi, Aveen Fattah Hajimam, Azam Rahmani, Leila Allahqoli, Gerhard Schmalz, Babak Nemat

**Affiliations:** ^1^ Department of Public Health, Social Determinants of Health Research Center, Research Institute for Health Development Kurdistan University of Medical Sciences Sanandaj Iran; ^2^ Department of Nursing, College of Nursing Alkitab University Altun Kupri Iraq; ^3^ Department of Midwifery, Nursing and Midwifery Care Research Center, School of Nursing and Midwifery Tehran University of Medical Sciences Tehran Iran; ^4^ Iran University of Medical Sciences (IUMS) Tehran Iran; ^5^ Department of Cariology, Endodontology and Periodontology University of Leipzig Leipzig Germany; ^6^ Health Network of Sanandaj Kurdistan University of Medical Sciences Sanandaj Iran

**Keywords:** behavior, beliefs, dental health, hemodialysis

## Abstract

**Objectives:**

Oral health‐related behaviors in hemodialysis patients are reduced, potentially leading to adverse consequences. The aim of the present study was to evaluate dental cleaning behavior (DCB) and related factors among hemodialysis patients.

**Methods:**

This cross‐sectional study was carried out in Sanandaj city, in the west of Iran in 2022. Using the census method, 115 hemodialysis patients from the dialysis center of Tohid Hospital were recruited. Data were gathered by a three‐section questionnaire. The first section included demographic characteristics, the second section involved variables related to the health belief model (HBM), and the third section assessed stages of DCB change based on the trans‐theoretical model. Analysis of data was done by assessing frequency, descriptive and inferential statistics such as *t* test, correlation coefficient, and regression.

**Results:**

Only 26.1% of participants reported DCB, defined as tooth brushing at least twice daily; 30.4% were in the precontemplation stage, 26.1% in contemplation, and 17.4% in the preparation stage. Perceived self‐efficacy was lower among patients who did not perform DCB. The odds of DCB increased with perceived self‐efficacy (odds ratio [OR] = 1.51, 95% confidence interval [CI] = 1.19–1.92, *p* < .05), cues to action (OR = 1.63, 95% CI = 1.03–2.55, *p* < .05), and decreased with perceived barriers (OR = 0.68, 95% CI = 0.47–0.97, *p* < .01).

**Conclusions:**

DCB of patients on hemodialysis requires improvement. Perceived self‐efficacy, cues to action, and perceived barriers constructs of the HBM should be considered in interventional programs targeting to increase oral health as well as in future research in the field.

## 1 INTRODUCTION

One of the systemic diseases that affects the tissues of the periodontium and the mouth is end‐stage renal disease (Queiroz et al., 2013). This situation starts with acute diseases such as diabetes and hypertonia (Webster et al., 2017) and if it does not cure, it becomes chronic, in which case it is called chronic kidney disease. At this stage, patients undergo hemodialysis (Queiroz et al., 2013). Studies have reported a relationship between hemodialysis and dental and oral diseases (Schmalz et al., 2020). In hemodialysis patients, oral infections can lead to sepsis and endocarditis. In addition, these patients are prone to dental and oral problems such as dental enamel problems, premature tooth loss, dry mouth, and periodontitis (Deschamps‐Lenhardt et al., 2019). On the other hand, periodontitis can be related to the development or worsening of kidney disease, as periodontal status has been identified as an indicator of the risk of developing kidney disease (Fisher & Taylor, 2009). Furthermore, the oral health situation is related to the morbidity and mortality of patients under dialysis (Misaki et al., 2019; Mizutani et al., 2020).

Alongside the relationship between kidney diseases or hemodialysis and oral health (Fisher & Taylor, 2009), the prevalence of behaviors related to dental and oral health is reduced in hemodialysis patients, respectively (Schmalz et al., 2016).

One study found that 40% of kidney hemodialysis patients did not brush their teeth regularly and 17% did not use any toothbrushes (Gürkan et al., 2008). Another study reported that 25.6% of chronic kidney disease patients did not use a toothbrush and 11.4% rarely brushed their teeth (Ruospo et al., 2014). The investigations emphasize providing oral care training to improve oral health behavior (Bharti et al., 2015; Shamsi et al., 2013). Despite dental and oral health education, most studies have not reported improvements in oral health function (brushing and flossing). One of its reasons is the lack of knowledge regarding the factors affecting dental cleaning behavior (DCB) (Al‐Hussaini et al., 2003; Sheiham & Watt, 2000). Furthermore, patients under dialysis were reported to show a kind of response shift in the perception of oral health conditions, potentially leading to reduced oral health behaviors; this phenomenon has been described for different patients with chronic diseases, including patients under dialysis as well as after transplantation (Schmalz et al., 2020). Some studies have reported on the factors affecting oral health behaviors in different groups of patients and have pointed out factors such as awareness, perceived severity, benefits, and barriers to oral health behaviors (Bahramian et al., 2017; Schmalz et al., 2016).

However, existing studies have not investigated the factors affecting DCBs in hemodialysis patients. The main question of this study is “what are the effective factors and the most important predictors of tooth cleaning behavior among hemodialysis patients based on health belief model (HBM)?”

Key elements of HBM focus on individual beliefs about health conditions, which predict individual health‐related behaviors. The model defines the key factors that influence health behaviors as an individual's perceived threat to sickness or disease (perceived susceptibility), the belief of consequence (perceived severity), potential positive benefits of action (perceived benefits), perceived barriers to action, exposure to factors that prompt action (cues to action), and confidence in the ability to succeed (self‐efficacy) (Jeihooni et al., 2019). This is important that reducing constructs of HBM, such as perceived self‐efficacy, benefits, and the perceived threat of dialysis individuals may cause terrible consequences such as loss of control and loss of the ability to live an independent life. Thus, that information would be useful to understand influential factors on the oral health behavior of patients on dialysis to develop individual, patient‐centered dental care approaches.

Accordingly, the aim of this study was to determine DCB and related factors based on HBM among hemodialysis patients. It was hypothesized that constructs of HBM might be considered in increasing DCB among hemodialysis patients.

## 2 STUDY POPULATION AND METHODS

This cross‐sectional study was conducted on dialysis patients in Sanandaj City, in the west of Iran, in 2022. The study was performed in full accordance with the Declaration of Helsinki, whereby the study protocol has been reviewed and approved by the local ethics committee (IR.MUK. REC.1397/374). Before inclusion, all patients were informed verbally and in writing about the study and gave their consent for participation. Every dialysis patient who was referred to Tohid Hospital and met the inclusion criteria was included in the study. Overall, 205 patients were screened, and a convenience sample of participants (*n* = 186) met the inclusion criteria, that is, the age range of 20–50 years, doing dialysis for more than 1 year, and the willingness to participate in the study, were consecutively recruited from the hospital. Patients suffering from diabetes, using alcohol and cigarette, patients with peritoneal dialysis, and patients with dentures were excluded from the study. Finally, 115 patients were included in the study and examined. The standard questionnaire comprised demographic characteristics, health beliefs, and DCB was used to collect the data. For this study, sufficient DCB was defined as using brushing teeth at least twice a day (Avenetti et al., 2020).

### 2.1 Primary outcome

Self‐reported DCB, the target outcome of this study, was derived from the stages of the DCB change questionnaire, which was based on the transtheoretical model of behavior change (Tilliss et al., 2003). The DCB comprises five stages, that is, precontemplation (people do not intend to take action in the predictable future), contemplation (people intend to start healthy behaviors in the foreseeable future, defined as within the next 6 months), preparation (people are ready to take action within the next 30 days), action (people have recently changed their behaviors, defined as within the last 6 months), and maintenance (people have sustained their behavior change for a while, defined as more than 6 months). The precontemplation, contemplation, and preparation stages indicate a lack of DCB, while the action and maintenance stages indicate that DCB has been adopted.

For this current study, a subject‐specific binary outcome for self‐reported DCB (no/yes) was derived from these five stages, indicating whether patients had not or had adopted DCB, that is, whether they were in the first three stages (precontemplation, contemplation, and preparation) or in the action and maintenance stages. The *κ* coefficient of the four items for stages of interdental cleaning behavior was 0.78 (Bahramian et al., 2017).

### 2.2 Primary predictor: Health beliefs

Health beliefs were derived from the HBM questionnaire for the prevention of dental caries (Karami et al., 2013). The 31‐item questionnaire comprises six conceptual dimensions, which were all considered as individual predictors. Those dimensions were: perceived sensitivity (six items, e.g., I have a high risk of dental caries), perceived severity (six items, e.g., I have an abnormal life when getting toothache), perceived benefits (four items, e.g., regular flossing can prevent of dental caries), perceived barriers (seven items, e.g., I do not have time for flossing), perceived self‐efficacy (six items, e.g., I can do flossing even if they are difficult), cues to action (two items, e.g., my family encourages me to floss my teeth), and external cues to action (five items, e.g., having a trainer helps me to do DCB to prevent dental caries). All items are rated on a 5‐point Likert scale from completely agree (score 5) to completely disagree (score 1). The validity and reliability of the instrument have been previously ascertained in the Iranian population (Karami et al., 2013).

### 2.3 Oral examinations

One experienced and calibrated dentist performed the oral examination, which included the gingival index of the participants. This index has three grades (grade 1: a slight change in gum color and texture, grade 2: gum redness, edema, and recession, and grade 3: severe inflammation and ulcer on gum) (Loe & Silness, 1963).

### 2.4 Study flow

After taking permission from the Kurdistan University of Medical Sciences and authorities of the hospital, explaining the study objectives, and taking informed written consent from the participants, data were collected. The participants were free to withdraw from the study at any stage, and confidentiality of the data was assured in all stages of the research. The researchers conducted standard face‐to‐face interviews in a quiet place. The administration of the questionnaire took 15 min to complete, and all the participants completed it.

### 2.5 Data analysis

SPSS (Version 23; SPSS Inc.) software was used for all statistical analyses. Logistic regression was applied to assess the association between DCB and health beliefs. Estimates were adjusted for age (≤45 years/>45 years), sex (women/men), job status (housewife/employed), income (≤US$200/>US$200), weight (≤65 kg/>65 kg), family history of dialysis (yes, no), duration of dialysis (≤4 years/>4 years), and gingival index (≤2/>2). Estimated logits and odds ratios (OR) were reported with corresponding 95% confidence intervals (95% CI). *χ* ^2^ and independent *t* test were used to assess group differences, and Pearson correlations were used to assess associations between HBM constructs based on research objectives. Statistical significance was established at *p* < .05.

## 3 RESULTS

### 3.1 Patient characteristics and DCB

A total of 115 patients with dialysis participated in the study. The mean age and gingival index of them were 46.3 ± 15.26 and 2.28 ± 0.6, respectively. About 74% of them were in the first stages of DCB change (30.4% were in the precontemplation stage, 26.1% in contemplation, and 17.4% in the preparation stage.), and 26% used dental cleaning tools regularly for more than 6 months (maintenance stage). Overall, 74% of patients (vs. 26%) did not perform DCB.

### 3.2 Comparison of DCB and HBM between subgroups

Table 1 shows patients with higher income were more likely to perform the behavior (*p* = .03). Also, people who did not have a government job performed less DCB (*p* = .01).

**Table 1:** Dental cleaning behavior (DCB) by demographic characteristics of the participants.

Demographic variables	DCB		
	Did not adopt DCB, *N* (%)	Doing DCB, *N* (%)	*p* Value (*χ* ^2^)
Age, years			
≤45	40 (70.2)	17 (29.8)	.36
>45	45 (77.6)	13 (22.4)	
Sex			
Woman	40 (69)	18 (31)	.22
Man	45 (79)	12 (21)	
Income, US$			
≤200	72 (78.3)	20 (21.7)	.03
>200	13 (56.5)	10 (43.5)	
Weight, kg			
≤65	41 (68.3)	19 (31.7)	.15
>65	44 (80)	11 (20)	
Job			
Unemployed	68 (80)	17 (20)	.012
Employed	17 (56.7)	13 (43.3)	
Family history of dialysis			
Yes	46 (71.9)	18 (28.1)	.57
No	39 (76.5)	12 (23.5)	
Duration of dialysis, years			
≤4	43 (76.8)	13 (23.2)	.49
>4	42 (71.2)	17 (28.8)	
Gingival index			
≤2	57 (78.1)	16 (21.9)	.17
>2	28 (66.7)	14 (33.3)	

*Note*: The significance level was set to 1% (0.01).

Table 2 presents the comparison of the mean and standard deviation of HBM constructs by the demographic characteristics of the participants. Perceived severity was higher in low‐income individuals (*p* = .03). The perceived benefits and cues to action were higher among employees and patients with higher education (*p* < .001). In patients with a family history of dialysis, perceived sensitivity (*p* < .001), perceived barriers (*p* = .04), and perceived self‐efficacy (*p* < .001) were higher and cues to action were low (*p* < .001).

**Table 2:** Comparison of mean and standard deviation of health belief model constructs by demographic characteristics of the participants.

Demographic variables	Means rank of determinants								
	Perceived sensitivity		Perceived severity		Perceived benefits	Perceived barriers	Perceived self‐efficacy	Cues to action	
Age, years									
≤45	16.82 (±1.86)		13.22 (±3.50)		12.38 (±2.95)	18.15 (±2.80)	16.40 (±4.19)	5.01 (±1.75)	
>45	16.29 (±2.24)		13.81 (±4.01)		12.68 (±2.62)	18.32 (±3.23)	15.53 (±3.72)	4.79 (±1.87)	
*p* Value^a^	.17		.40		.56	.76	.24	.50	
Sex									
Woman	16.53 (±1.95)		13.25 (±3.10)		12.79 (±2.43)	18.01 (±2.76)	16.48 (±4.20)	5.10 (±2.03)	
Man	16.57 (±2.20)		13.78 (±4.34)		12.28 (±3.10)	18.47 (±3.26)	15.43 (±3.67)	4.70 (±1.54)	
*p* Value^a^	.90		.45		.32	.42	.16	.23	
Income, US$									
≤200	16.44 (±2)		13.90 (±3.64)		12.46 (±2.67)	18.33 (±2.87)	15.79 (±3.83)	4.73 (±1.67)	
>200	17 (±2.33)		12 (±3.93)		12.82 (±3.25)	17.86 (±3.59)	16.65 (±4.48)	5.56 (±2.19)	
*p* value^a^	.25		.03		.58	.50	.35	.10	
Weight, kg									
≤65	16.73 (±1.94)		13.88 (±4.08)		13.20 (±2.56)	18.13 (±3.08)	16.18 (±4.51)	5.05 (±1.69)	
>65	16.36 (±2.20)		13.12 (±3.38)		11.81 (±2.85)	18.36 (±2.96)	15.72 (±3.29)	4.74 (±1.93)	
*p* Value^a^	.34		.28		.007	.68	.54	.37	
Job									
Housekeeper	16.63 (±2.02)		13.49 (±3.54)		12.11 (±2.74)	18.18 (±2.86)	15.85 (±3.73)	4.44 (±1.57)	
Employed	16.33 (±2.21)		13.60 (±4.39)		13.60 (±2.59)	18.40 (±3.47)	16.26 (±4.63)	6.20 (±1.82)	
*p* Value^a^	.49		.89		.006	.74	.63	.001	
Family history of dialysis									
Yes	16.84 (±1.99)		13.54 (±3.40)		12.89 (±2.52)	18.59 (±2.71)	15.70 (±3.91)	4.82 (±1.76)	
No	16.19 (±2.13)		13.49 (±4.21)		12.09 (±3.05)	17.80 (±3.34)	16.29 (±4.05)	5 (±1.87)	
*p* Value^a^	.001		.87		.93	.04	.001	.001	
Duration of dialysis, years									
≤4	16.60 (±2.38)		13.01 (±3.62)		12.62 (±2.64)	18.07 (±2.42)	15.67 (±3.94)	4.96 (±1.86)	
>4	16.50 (±1.74)		14 (±3.86)		12.45 (±2.93)	18.40 (±3.50)	16.23 (±4.01)	4.84 (±1.76)	
*p* Value^a^	.80		.16		.74	.55	.45	.73	
Gingival index									
≤2	16.50 (±2)		13.79 (±2.88)		12.83 (±2.54)	18.30 (±2.81)	15.45 (±3.52)	4.80 (±1.760)	
>2	16.64 (±2.21)		13.04 (±4.95)		12.02 (±3.12)	18.14 (±3.37)	16.85 (±4.55)	5.07 (±1.89)	
*p* Value^a^	.73		.37		.13	.78	.08	.45	

^a^ Independent‐samples *t* test.

Table 3 shows the mean and standard deviation of the variables of the study. The mean score of cues to action (*p* < .001), perceived benefits (*p* < .01), and perceived self‐efficacy (*p* < .001) were higher in the higher stages of behavior change.

**Table 3:** Comparison of mean and standard deviation of the gingival index, perceived self‐efficacy, barriers, benefits, cues to action, perceived sensitivity, and perceived severity by patients' stages of dental cleaning behavior.

Stages of behavior change	Gingival index		Perceived Self‐efficacy		Perceived barriers		Perceived benefits		Cues to action		Perceived sensitivity		Perceived severity	
	Mean	SD	Mean	SD	Mean	SD	Mean	SD	Mean	SD	Mean	SD	Mean	SD
Precontemplation	2.28	0.572	16	3.44	18.97	3.73	11.54	2.86	4.51	1.65	16.11	2.13	14.68	4.67
Contemplation	2.1	0.54	14.06	1.04	18.66	1.93	12.86	2.3	3.9	0.88	16.73	2.28	12.43	2.11
Preparation	2.45	0.6	13.7	1.86	17.35	3.34	12.55	2.92	4.5	1.76	16.9	1.88	12.95	3.41
Action	2.35	0.63	19	5.34	17.14	3.15	12.28	2.75	6.57	1.86	17.5	1.99	13.85	3.97
Maintenance	2.37	0.71	19.62	4.96	17.93	1.91	14.31	2.6	6.62	1.4	15.93	1.52	13.43	3.89
*p* Value	.3		.001		.17		.01		.001		.15		.16	

### 3.3 Correlation matrix of HBM parameters

Table 4 illustrates the results of the correlation matrix between independent variables. Perceived self‐efficacy was directly correlated with cues to action (*p* < .01). Also, cues to action with perceived barriers, benefits, and perceived severity had a statistically reverse correlation with perceived social support (*p* < .01) and perceived benefits (*p* < .05). Perceived benefits had a statistically reverse correlation with perceived barriers (*p* < .01).

**Table 4:** Correlation matrix of health belief model constructs (*n* = 115).

Variables	Perceived sensitivity	Perceived severity	Perceived benefits	Perceived barriers	Perceived self‐efficacy	Cues to action
Perceived sensitivity	1					
Perceived severity	0.095	1				
Perceived benefits	−0.001	0.408**	1			
Perceived barriers	0.027	0.380**	−0.319**	1		
Perceived self‐efficacy	−0.001	0.109	0.137	0.100	1	
Cues to action	−0.030	0.123	0.290**	−0.033	0.437**	1

** Correlation is significant at the .01 level (two‐tailed).

### 3.4 Regression analysis

Based on the results of regression analysis related to behavior, some variables such as self‐efficacy, perceived barriers, and cues to action remained in the regression and their significant relationships to behavior were confirmed (Table 5). The odds of DCB increased with perceived self‐efficacy (OR = 1.51, 95% CI = 1.19–1.92, *p* < .05), cues to action (OR = 1.63, 95% CI = 1.03–2.55, *p* < .05), and decreased with perceived barriers (OR = 0.68, 95% CI = 0.47–0.97, *p* < .01).

**Table 5:** Logistic regression parameter estimates.

Predictors	*b*	SE	Odds ratio	95% CI	Wald	*p* Value
*Model*						
Age, years	−0.222	0.768	0.801	0.17–3.61	0.08	>.05
≤45						
>45						
Sex	0.311	0.800	1.364	0.28–6.55	0.15	>.05
Woman						
Man						
Income, US$	0.487	1.278	1.628	0.13–19.94	0.14	>.05
≤200						
>200						
Weight, kg	−0.446	0.798	0.640	0.13–3.06	0.31	>.05
≤65						
>65						
Job	−0.983	1.159	0.374	0.03–3.62	0.71	>.05
Housekeeper						
Employed						
Family history of dialysis	−1.188	0.878	0.305	0.05–1.70	1.83	>.05
Yes						
No						
Duration of dialysis, years	0.718	0.766	2.050	0.45–9.20	0.87	>.05
≤4						
>4						
Gingival index	0.521	0.813	1.683	0.34–8.28	0.41	>.05
≤2						
>2						
Perceived sensitivity	0.105	0.194	1.111	0.75–1.62	0.29	>.05
Perceived severity	−0.030	0.116	0.971	0.77–1.21	0.06	>.05
Perceived benefits	0.011	0.196	1.011	0.68–1.48	0.003	>.05
Perceived barriers	−0.380	0.181	0.684	0.47–0.97	4.39	<.05
Perceived self‐efficacy	0.418	0.121	1.519	1.19–1.92	11.86	<.05
Cues to action	0.484	0.231	1.623	1.03–2.55	4.41	<.05

*Note*: Variable dependent: dental cleaning behavior.

Model (likelihood ratio) *χ* ^2^ = 76.77, *df* = 16, *p* < 0.01.

Negelkerke *R* ^2^ = 71.3%.

Percent correctly classified = 90.4%.

Abbreviation: CI, confidence interval.

## 4 DISCUSSION

The present study was conducted to investigate the state of DCB and related factors among hemodialysis patients in Sanandaj City. The results of the study showed that the cues to action, perceived self‐efficacy, and perceived sensitivity were higher in patients with a family history of dialysis and perceived barriers were lower in them. Acheson et al. (2010) reported that a family history of the disease was associated with perceived risk and worry of the disease (coronary heart disease, stroke, diabetes, and breast, ovarian, and colon cancer) and made the disease more acute but a family history of the disease was not related to perceived control regarding the disease. Hwang et al. (2019) also showed that men with a family history of cancer performed better and had more health‐related behaviors. Probably, observing the severity of the disease in the people around them, the severe social, psychological, and physical effects of the disease on them, and the fear of negative consequences are among the factors that have encouraged patients with a family history to behave and obtain information.

The current study's results showed that the mean score of the cues to action and perceived benefits were higher among employees. Kiyak (1988) states that people with higher education and income are more willing to absorb training, understand the benefits of behavior, and have a favorable attitude toward maintaining oral health. Probably, employees who have education not only analyze and understand more appropriately the issues related to behavior but also have a greater desire to learn to acquire skills. Obtaining information from colleagues, receiving encouragement from them to behave and comply with health tips, and the presence of appropriate and sufficient social support and relationships in the work environment can be among the factors related to a high guide score for action, social support, and perceived benefits.

The results showed that almost three‐fourths of the patients were in the initial stages (precontemplation, contemplation, and preparation stages). Thereby, patients did not only show reduced DCB, the average score of perceived benefits and self‐efficacy was lower in them, too. Emani et al. (2016) showed that the perceived self‐efficacy score and perceived benefits are higher in the final stages of the behavior. According to the transtheoretical theory, self‐efficacy and perceived benefits as well as social support are higher in the final stages of behavior (Sardi et al., 2019). Jamieson et al (2014) and Silk et al. (2008) also showed that low self‐efficacy is related to worse oral health. Ahmed et al. (2019) also reported that a decrease in self‐efficacy causes a decrease in behaviors related to oral health and increases oral diseases. Based on the principles of Bandura's (1977) social‐cognitive theory, people with high self‐efficacy observe the standards of behavior and do it more. According to the principles of this theory, people with self‐efficacy are less afraid of changing their behavior or more confident with being successful with a behavioral change. On the other hand, when a person succeeds in performing the behavior, his self‐efficacy also increases (Bandura, 1977). Probably, the reduction of depression, stress, and anxiety in dialysis patients, appropriate role models from others, previous experiences of the individual, strengthening of beliefs and motivations, purposefulness in behavior and focus on small short‐term goals, increasing social support, especially the support of family and hospital staff, can increase the self‐efficacy of dialysis patients to perform behaviors related to oral health.

The results of this current study showed that the cues to action in the initial stages of behavior are lower than that in other stages. Based on the theoretical foundations of the HBM, cues to action are higher in people who practice preventive behaviors than in other people (Glanz et al., 2002). Social learning theory also considers social supports to be effective in performing the behavior (Macintosh, 1982). Probably, the participants who are in the initial stages of the behavior did not receive the necessary encouragement, support, and companionship from family, friends, and the media to continue the behaviors related to dental health. On the other hand, people in the thinking stage may feel that they receive high social support while performing the behavior, but when people perform the behavior in the real world, the meaning of social support may change from their point of view. Perceived social support is more important for patients than real social support. Maybe during dialysis therapy, the perception of social support changes, because social support might be adapted as “normal.”

Variables of self‐efficacy, cues to action, and perceived barriers were predictors of DCB in hemodialysis patients. Based on the theoretical foundations of Pender's et al. (2010) model of health promotion, the presence of obstacles causes not to perform the behavior and stop it. Bharti et al. (2015) reported some barriers to oral health such as age, fear, and anxiety caused by bleeding gums and dental examination, socioeconomic status, low income, lack of knowledge of the time and place of providing dental services, and improper communication. Probably physical problems and complications of kidney disease, low attitude toward oral health and behavior, lack of insurance and access to dental services, fear of care, lack of social support, high treatment costs, bleeding and gum infection, lack of health‐related support policies, low understanding of the benefits of oral health, low understanding of the severity of oral diseases and self‐efficacy, and the lack of specific training to promote oral health to patients can be barriers of performing behaviors related to oral health in dialysis patients. Paying attention to all the above matters can be useful in improving the oral and dental health of hemodialysis patients.

## 5 STRENGTHS AND LIMITATIONS

The strengths of this study should be highlighted including the first study on DCB related to transtheoretical model and health beliefs, a large cohort in a difficult patient group, and the practical relevance of the question. A cross‐sectional design, no information on causality is available, strict in‐ and exclusion criteria limiting the generalizability of the sample, and lack of comparison group were the limitations of the present study. Several additional pieces of information would have been of certain interest to interpret the data. Those include periodontal status and sociodemographic data of the participating patients, as these could be influential factors on the examined parameters. Overall, a healthy control group would have been helpful to interpret the oral hygiene behavior of the patients; however, this study primarily focused on patient‐centered variables, which is also of high interest within the group of hemodialysis patients, irrespective of differences with healthy control.

## 6 CONCLUSION

DCB of patients on hemodialysis was reduced, as it was adequately performed by only one‐quarter of patients. Thereby, self‐efficacy, cues to action, and perceived barriers were related to DBC, making those factors a reasonable approach to improve the DBC and thus oral health in those individuals. Future dental care concepts and related research should consider those issues, alongside the stage of the patient according to the transtheoretical model.

## AUTHOR CONTRIBUTIONS

Arezoo Fallahi and Babak Nemat designed and made the concept of the research, and wrote the manuscript. Aveen Fattah Hajimam, Azam Rahmani, and Leila Allahqoli analyzed data, and Gerhard Schmalz edited the manuscript.

## CONFLICT OF INTEREST STATEMENT

The authors declare no conflict of interest.

## Data Availability

The data that support the findings of this study are available from the corresponding author upon reasonable request.
